# Cure of a Confirmed Opium-Eater

**Published:** 1867-02

**Authors:** E. Andrews

**Affiliations:** Professor of Surgery in Chicago Medical College; Chicago


ARTICLE IX.

### CURE OF A CONFIRMED OPIUM-EATER.—RECORD OF THE SYMPTOMS.

By E. ANDREWS, M.D., Professor of Surgery in Chicago Medical College.

I do not remember to have seen anywhere an accurate record of the symptoms which follow when an opium-eater breaks off suddenly from his pernicious indulgence. I am induced, therefore, to present the following case from my practice, believing that it will be instructive to the profession:—

R. G———, aged about 35 years, of good general habits and character, sent for me to his house, and informed me that he was addicted to the use of opium, and desired to break off the habit. He stated that the appetite was acquired from the long-continued prescription of anodynes by his physician, some two years ago, during a painful sickness. Since then his tolerance had gradually increased, until his present allowance was ten grains of sulphate of morphia a-day, which he took in two doses. He informed me that he had no love for it, and had got past deriving any pleasure from its use; his daily allowance merely serving to keep him out of an intense but vague feeling of wretchedness, which seized upon him when he attempted to go without his doses. His countenance presented a slightly haggard and nervous aspect, but otherwise there was no appearance of physical or mental deterioration. He was very apprehensive, as most opium-eaters are, that total abstinence from his drug would kill him. I assured him that he would be very miserable for a few days, but that he would not die. I accordingly directed him to stop at once, and took his word of honor, to use no opiate unless I ordered it; I promised to watch him closely, and that if he incurred any danger of dying I could easily prevent that by giving some small doses of morphine, at the proper time. I further told him that he might as well die as be an opium-eater. I then removed all opiates from his house, and prescribed some pills of quinine, cannabis indica and hyoscyamus to occupy the attention of his nervous system. The following is the history of the subsequent days:—

*1st Day.*—Found him feeble, restless, very weak, and unable to get out of bed. Had no sleep, terribly restless, at times delirous, but though more generally rational. Has severe pains occasionally in the sternum, and the lower part of the spine, but the chief distress is an intolerable, undefinable "misery." His pulse is 95, and very soft and weak. His determination to persevere holds out well. Has frequent vomiting and some diarrhœa. The pills seem to be totally powerless to ease him. Gave a few inhalations of ether without any increase of comfort; potations of whiskey or brandy are vomited up. Every effort to find a substitute for the opiate seems almost a total failure. Oyster soup for nourishment.

*2d Day.*—Mind slightly wandering in the morning, but rational the rest of the day. Vomits occasionally, and has slight diarrhœa. Pulse 68, and stronger. Milk punch with wine and crust coffee for nourishment. Cannot retain solid food. No medicine.

*3d Day.*—Mind wandering again in the morning. Pulse 66, but very feeble. Countenance shrunk. Some vomiting and diarrhœa. Feeling uneasy lest his prostration should go too far, I gave him three small doses of laudanum combined with hyoscyamus and cannabis indica. He revived so rapidly and decisively that I was satisfied that he was in no danger, and forbade any more to be given. Beef-tea with wine and crust coffee.

*4th Day.*—Morning. Slept five hours for the first time. Rational; no vomiting nor purging. Unable to sit up yet. Tincture of cinchona, hyoscyamus, and cannabis indica.

*Afternoon.* More prostrate. Hitherto his courage has kept up, and he has not asked for morphine. Now he is broken down and begs for it. Gave one small dose which exhilirated him surprisingly, forbade any more.

*5th Day.*—No vomiting, but an exhausting diarrhœa. Tried to check it with astringents, terebinthinates, etc., without success. Was obliged to add a little opium, which accomplished the purpose and greatly revived him.

*6th Day.*—Diarrhœa worse again; checked it with pills containing large doses of nitrate of silver, and small doses of opium. Appetite improves.

*7th Day.*—Diarrhœa returned and was checked again by the same pills. Feels better; sits up in bed.

*8th Day.*—Improving. No medicine.

*9th Day.*—Improving, dressed, and lying on a sofa. Tincture of nux vomica as a tonic.

*11th Day.*—Doing finely; the nux vomica has produced a great increase of muscular tone, eats well. No diarrhœa. Stopped the medicine.

*13th Day.*—Improving. Walks about the house a little. No medicine.

*15th Day.*—Doing finely. Has no desire for opium, feels perfectly comfortable, though weak. The tongue, which the first few days had a dense white fur, is now clean. Advised him on no account to take any opiate, nor to prop up his debility by substituting alcoholic drinks, lest he merely exchange one bad habit for another, but to recover the balance of his strength by the vigor of his own constitution. Discharged from treatment, apparently cured of his appetite.

